# Update on bioethical, medical and fertility issues in gender incongruence during transition age

**DOI:** 10.1007/s40618-023-02077-5

**Published:** 2023-04-18

**Authors:** A. C. Conflitti, M. Spaziani, F. Pallotti, M. G. Tarsitano, A. Di Nisio, D. Paoli, F. Lombardo, TALENT Study Group

**Affiliations:** 1 Laboratory of Seminology-Sperm Bank “Loredana Gandini”, Department of Experimental Medicine, “Sapienza” University of Rome, Viale del Policlinico 155, 00161 Rome, Italy; 2 Department of Experimental Medicine, Section of Medical Pathophysiology, Food Science and Endocrinology, Viale del Policlinico 155, 00161 Rome, Italy; 3 Department of Medical and Surgical Science, University Magna Graecia, Catanzaro, Italy; 4 Department of Medicine, Operative Unit of Andrology and Medicine of Human Reproduction, University of Padova, Padua, Italy

**Keywords:** Gender affirming hormone treatment, Fertility preservation, Gender identity, Gender incongruence, Gender dysphoria

## Abstract

**Purpose:**

Many issues still remain unresolved in the management of pubertal patients with gender incongruence (GI). The aim of this review is to discuss the main aspects of the treatment of these patients to provide a practical approach for clinicians.

**Methods:**

A comprehensive literature search within PubMed was performed to provide updates of available evidence regarding the impact on bioethical, medical and fertility issues in gender incongruence during transition age.

**Results:**

Gender Affirming Hormone Treatment (GAHT) and Gender Affirming Surgery (GAS) can induce unsatisfaction with change, future regrets, and the risk of infertility. This raises ethical issues especially in the management of pubertal patients that remain unresolved. Therapy with GnRH analogues (GnRHa) is intended to delay puberty, so as to give the adolescent a longer period of time to decide whether to continue with the treatments. At the level of physical changes, this therapy may have an effect on bone mineralization and body composition; however, long-term longitudinal data are not yet available. An important feature related to the use of GnRHa is the risk of fertility. Gamete cryopreservation is the most established method of fertility preservation (FP) and should be counselled to transgender adolescents. However, these patients are not always interested in having biological children.

**Conclusion:**

Based on the current evidence, there is a need to conduct further research to clarify certain issues and to standardize clinical practice and improve counselling in transgender adolescent decision making and avoid regrets in the future.

## Introduction

Nowadays, gender identity and gender incongruence (GI) are frequently discussed and accepted concepts in most of Western societies [1]. Gender identity is the identification of a person of being male, female, or neither/both [2]. In particular, considering a non-binary, a continuum from female-to-male, the subjects with GI are up to 10% [3, 4]. GI is defined as a condition in which the gender identity does not match with the assigned sex at birth. In addition, gender dysphoria (GD) is when GI is associated to discomfort or distress [2] and has a prevalence between 0.6 and 2.7% in childhood and adolescence [5]. Anyway, not all patient, especially children, present GD, but only GI [6], while, in puberty, GD is higher and could be associated to depression and suicidal ideation [7]. In general, GD is increasing in the last decades [8] and the causes could be found in the changes of society acceptance, a greater awareness in healthcare professionals and an increased information available in social media [9].

The basis of GI development is still unknown. In the past, the causes were indicated in family dynamics such as the absence of the father or in the mother–child interaction [10] and subsequently in a multiple (child, parents and environment) factors process emerging simultaneously [11]. Nowadays, genes, hormones, brain structure and behaviour seem to be all contributor of GI, but without a clear interaction and sequences [1].

In particular, specific genes are not identified but genetic contribution is demonstrated thanks to twin studies in which concordance of GI was found in monozygotic twins, but not in dizygotic [12]. The role of hormones is identified in specifically modification of testosterone (T), high level of T in prenatal female and low T in natal male seem acting on brain development and directing to GI [13, 14]. The brain remodelling is another important aspect of GI occurrence, GI brains show similitudes with their identify gender and not with the assigned one [15].

Puberty represents a crucial point in the life of every individual. The hormonal changes and the secondary sexual characteristics radical modify ourselves and ambient perceptions [16]. For someone, such as some non-binary individuals, is so hard that they prefer to maintain an androgynous gender expression with improvement of mental health but remain the ethical problem of this treatment [17].

The puberty incoming for the patient with GI could be announced by (1) a childhood with an acceptance of birth gender, (2) a childhood with an internal tension or (3) a childhood characterized by a clear known GI [18]. For some adolescents the history of GI begins in the childhood, for others after the pubertal changes or in the adolescence [19]. So, the history of each patient is different, and they need specialized multi-professional team. Despite the presence of experts, numerous questions, principally ethical, remain open. Surely, when a pubertal patient with GI and the parents arrive in a specialistic clinic, a complete evaluation and prospect have to be provided [20].

Therefore, in this review we aim to discuss the main aspects of the treatment of the pubertal patients with GI to provide a practical approach for clinicians involved in this issue.

## Material and methods

To discuss the main issues regarding gender incongruence during the transition age, a narrative review was carried out in PubMed on July 2022. The database search was performed using the following terms for each paragraph:

- Bioethical consideration: Gender, youth, adolescence, ethics, bioethics, ethical issue;
- Pubertal suppression: GnRH analogues, bone mineral density, Tanner stage, puberty;
- Fertility preservation: Fertility, preservation, transgender, adolescence, youth, sperm cryopreservation.

All papers that addressed the issues of interest were considered, with no restrictions on publication date given the actuality of the topic. The reference list was screened to determine inclusion or exclusion. Firstly, reviews, meta-analyses, and papers published in a language different from English were excluded from the search. The remaining publications were subjected to full-text screening to determine the pertinence using the following inclusion criteria:

- Publications that present an ethical issue related to management of transgender in transition age;
- Publications that dealt with puberty, the treatments available to stop puberty in adolescent patients, and their effects;
- The publication discusses transgender youth's desire for parenthood, fertility counselling and the options of preservation.

In conclusion, all relevant peer-reviewed publications in English language were considered. Other relevant papers were selected from the reference list.

## Results

The publications included in this review are represented in the tables below. For ethical aspect 3 publications were considered (Table 1), for pubertal suppression 20 publications were included (Table 2), and for fertility preservation 29 publications were included (Table 3). Other related papers useful in explaining topics, such as guidelines and position statements, were added to these results.

**Table 1:** Publications related to ethical issues

Topic	Contents	References
Gender Dysphoria: Bioethical Aspects of Medical Treatment	A review on general ethical aspects of transgender medicine (from adolescent treatment, to fertility issues and risk of regret after gender reassignment)	Bizic MR et al. 2018 [23]
Moral Challenges in Transgender Care: A Thematic Analysis Based on a Focussed Ethnography	A thematic content analysis of multi-disciplinary meetings and psychodiagnostics assessments that identified key challenges (eligibility criteria, treatments, role of clinical guidelines and decision-making issues)	Gerritse et al. 2018 [24]
Two dilemmas for medical ethics in the treatment of gender dysphoria in youth	A focus and debate on challenges faced by families and healthcare providers in the management of children presenting with GD: the informed consent and the best timing for gender-affirming procedures	Baron et al. 2022 [26]

**Table 2:** Publications related to pubertal suppression

Topic	Contents	References
GnRHa formulations most frequently used	Triptorelin and Leuprorelin, at a dosage of 3.75 mg every 28 days; if the GNRHa is not practicable (e.g. too expensive): oral or injectable progestin formulations may be used	Cohen-Kettenis PT et al. 2011 [35] Vlot MC et al. 2017 [36] Schagen SE et al. 2016 [37] Schneider MA et al. 2017 [38] Coleman E et al. 2022 [19]
When to start GnRHa	At the discretion of the MHP and endocrinologist, in case of: severe psychological impairment; absence of clinical, psychological or social conditions that may interfere with the adherence to the therapy; Tanner G2/B2	Hembree WC et al. 2017 [41]
Benefit of treatment	Better physical outcome; improved global functioning; reduction in: suicidal ideations, depression, psychological distress, binge drinking and drug abuse	Panagiotakopoulos L et al. 2020 [42] Turban JL et al. 2020 [43] de Vries AL et al. 2014 [44]
Consequences of GnRHa on bone health	Absence of significant changes in BMD after 3 years of treatment	Joseph T et al. 2019 [48]
	Improvement of lumbar BMC and BMD after 24 months of GnRHa	Carmichael P et al. 2021 [49]
	Stabilization or small decrease of BMAD; BMAD z-scores decrease	Schagen SEE et al. 2020 [50] Klink D et al. 2015 [51] Stoffers IE et al. 2019 [52]
Consequences of GnRHa on body composition	Increased fat ratio in AMAB; Decreased fat ratio in AMAB	Klaver M et al. 2018 [53]
	Increase in fat mass and decrease in lean body mass percentage	Schagen SE et al. 2016 [37]
	No change in body composition	Klink D et al. 2015 [51]
Consequences of GnRHa on fertility	Scarce maturation of the gametes, which requires the cryopreservation procedure	Cheng PJ et al. 2019 [54] Burns KC et al. 2018 [55] Rew L et al. 2021 [56]

**Table 3:** Publications related fertility preservation

Topic	Contents	References
General features of Fertility Preservation (FP)	Gender Affirming Hormone Treatment could have an impact on fertility, so FP is recommended before starting therapy	Leung et al. 2019 [59] Fisher et al. 2022 [58] Coleman et al. 2022 [19]
Oocyte cryopreservation	Oocyte cryopreservation is an effective method to preserve fertility of youth transgender men. The GnRHas have an impact on gamete maturation but do not cause permanent damage to gonadal function, so an interruption of testosterone can result in the recovery of ovarian function. However, the long-term effects of therapy are not known, so it is recommended to do FP before therapy	Light et al. 2014 [63] Duncan et al. 2017 [67] Maxwell et al. 2017 [65] Rothenberg et al. 2019 [66] Amir et al. 2020a [68] Amir et al. 2020b [70] Armuand et al. 2020 [62] Marschalek et al. 2020 [69] Barrett et al. 2022 [64] Adeleye et al. 2019 [61]
Semen cryopreservation	Semen cryopreservation is the method of FP for young transgender women. GAHT can cause alteration of seminal sperm parameters. Recovery of spermatogenesis is possible after treatment discontinuation, but neither the recovery time nor the effects of therapy on offspring are known	Adeleye et al. 2019 [71] Barnard et al. 2019 [72] Sermondade et al. 2021 [60]
Use of FP among transgender youth, barriers to the use of FP for transgender youth and their desire of parenthood	It is observed low adherence to fertility preservation. The reason is related to barriers such as cost, inadequate access to medical care, inconvenience in retrieving gametes, and concern in delaying treatment	Chen et al. 2017 [75] Nahata et al. 2017 [76] Kyweluk et al. 2018 [80] Chiniara et al. 2019 [73] Morrison et al. 2020 [74] Persky et al. 2020 [81] Kerman et al. 2021 [57] McCallion et al. 2021 [77] Boguszewski et al. 2022 [78]
How to improve the clinical practice	Providers suggest that the presence of a disciplinary team, providing written information, personalizing the approach and developing a decision aid programme could improve clinical practice	Johnson et al. 2016 [86] Chen et al. 2018 [82] Chen et al. 2019 [85] Lai et al. 2021 [83] Quain et al. 2021 [84]

### Bioethical considerations

An important challenge that the healthcare professional faces in the management of the transgender patient is posed by bioethical issues. The already complex network of social rules, laws, standards of medical care is made increasingly difficult to manage due to heterogeneity in subjective goals in gender reaffirming that are claimed by each transgender person. Gender transition may have a social component (“social” transition) that involves the conduction of the role of the wanted gender, and a medical dimension. The Gender Affirming Hormone Treatment (GAHT) requires a team of experienced professionals and is the necessary step to achieve various degrees of phenotypical changes. Some subjects may decide to proceed further by radically and permanently change through a Gender Affirming Surgery (GAS).

A first element to take into consideration is the currently fluid and still evolving public awareness of Gender Incongruence, which inevitably impacts on both regulations and transgender healthcare. The relatively recent de-pathologization of transgender identities, strengthened by the introduction of the 11th revision of the International Classification of Diseases, has promoted an invaluable level of engagement of the transgender and gender diverse people in their health care management and drives towards more socially inclusive policies and laws to improve access to treatments and social inclusion [19, 21]. This, in general, is especially imperative for health care workers who require to include knowledge of gender-affirming interventions to reduce disparities and even possible harms [22].

Even so, as all interventions (GATH and GAS) may have side effects, induce an unsatisfactory level of changes or cause permanent sterility, other major ethical challenges refer to the management of subjects in their transition age and the risk of infertility, also considering the possibility of regret after irreversible surgical procedures [23].

A relevant risk is that gender incongruent subjects may not always achieve a clear perception of treatment consequences, even after being sufficiently informed since the relation with the healthcare professional may be severely hampered by a range of circumstances (from mental and medical concerns to cultural pressure and socio-economic issues) causing the lack of a strong shared basis for a medical/surgical decision making [24].

This may assume an even greater importance when the healthcare professional need to manage a subject in his transition age. Major debates have been raised on whether is ethical to treat an adolescent with signs of gender incongruence who may physiologically evolve into a cis-gender subject and the capability of expressing a truly informed consent [25]. The latter is a critical aspect since the acquisition of an informed consent and the subsequent clinical/therapeutical actions require a comprehensive evaluation of the subject’s emotional, cognitive, and psychosocial maturity, which may vary among individuals in the transition age [19]. The adolescent must be able to understand the treatment, its benefits and potential risks, as well as the short and long-term consequences. Furthermore, depending on specific countries laws, the parents/legal guardians are those who legally provide the consent and must be fully included in the process of informed consent acquisition.

There is also need to consider if the clinical and social background of GI justifies the possible effects on the adolescent’s health (especially in regards to future fertility) and whether the lack of treatment may cause even more serious issues (for example in case of dysphoric children with mental health comorbidities) [26]. Puberty processes advancing in an adolescent with GI may introduce irreversible changes and from an ethical perspective cannot be considered a neutral action as it may create potentially harmful effects for the transgender young person [19].

Nonetheless, despite the relative safety of hormonal treatments, debates on these issues are still ongoing and until further research is conducted these ethical concerns remain unanswered.

### **Pubertal suppression: GnRH analogues**

The use of GnRH analogues (GnRHa) is historically approved in some well-defined pathological conditions, such as central precocious puberty, and in the treatment of specific causes of short stature. For some years now, the use of puberty-suppressing medication has also been approved in the setting of gender-affirming healthcare services, to reduce the psychological trauma that patients suffering from GD or GI may experience, as they see their body maturing towards a sex in which they do not identify [27].

All the clinical modifications that occur during puberty are the direct consequence of the hypothalamic-pituitary–gonadal axis (HPG) activation, involving an increase in gonadotropin-releasing hormone (GnRH) pulsatility and, consequently, in gonadotropins and gonadal steroids. The normal duration of puberty is about 5–6 years [28].

In females, puberty normally starts between the age of 8.5 years old and 12.5 years old (on average 10.5 years old), and the first modification is represented by the so-called telarche [29]. The beginning of puberty in males is normally later than in females, namely between 9.5 and 13.5 years old (on average 11.5 years old), and the first sign is the testes volume increase at 4 mL [30].

GnRHa stop the progression of puberty through the desensitization of the GnRH receptor, consequently suppressing the secretion of the gonadic steroids [31]. Some recent studies seem to demonstrate an additional mechanism of action of puberty-suppressing medication, which consists in inducing sustained increased levels of the free α-subunit of the GnRH membrane receptor, which causes a dysfunction of the receptor itself [32]. This mechanism of action would explain the evidence, in other studies, of a receptor desensitization of only 30% compared to basal values, highlighting a possible dual mechanism of action [33].

There are several types of GnRHa, but the most frequently used in clinical practice are those administered monthly, specifically every 28 days, known as monthly depot GnRHa. There are, however, other formulations, ranging from 3-monthly (12-week) to 6-monthly (24-week) administrations. Finally, there are also subcutaneous histrelin implant, which require a small surgical procedure for their placement, and are able to release their active ingredient in a gradual manner, without the need for periodic injections [34]. Anyway, in the studies carried out on the suppression of puberty in the context of GD or GI, the typology of formulations most commonly used are triptorelin and leuprorelin, at a dosage of 3.75 mg every 28 days [35–38]. The purpose is to dilate the diagnostic phase, to give the adolescent a longer period of time to decide whether to face the stage of irreversibility of the change of sex [39]. One of the main features of the therapy, is given by the reversibility of the intervention, which allows to resume the normal course of puberty in the event that the adolescent no longer expresses the desire for a transition of sex [40].

#### GnRHa treatment in adolescents with GD/GI

The latest Endocrine Society clinical practice guidelines indicate that GnRHa therapy is feasible in adolescents with GD/GI when the mental health physician (MHP) determines the degree of severe psychological impairment related to an intense and prolonged pattern of gender noncompliance or GD [41]. The guidelines also stress the need for evidence of a significant worsening of the subject, after the pubertal onset, and the absence of clinical, psychological or social conditions that could compromise the onset and adherence to the block therapy. It is mandatory the complete subject’s awareness regarding the typology of therapy to which he/she will be subjected, its duration, and the possible adverse effects: the adolescent, in this way, should therefore be able to lend his full informed consent to the performance of the puberty-suppressing medication. In addition to the role of MHP, the one of the paediatric endocrinologist is absolutely relevant, since in addition to agree with the goodness of the GnRHa treatment, must confirm the absence of contraindications, and determine the exact moment when to start it. In this respect, is essential a thorough physical examination, to detect a pubertal stage of Tanner G2/B2, reached which the beginning of therapy is possible. Actually, as previously reported, the young adolescent should be able to observe the initial sexual maturation towards the opposite sex than the desired one, to assess the possible/probable psychological worsening, which would be the further proof of the fairness of the therapy with GnRHa. To note, the guidelines are for “suggestions”, but not for “recommendations”, given the low-quality evidence. It is still recommended, where indicated, the use of GnRHa to suppress pubertal hormones [41].

#### Advantages of therapy with GnRHa

The most significant advantages are a better physical outcome, and the avoidance of the violent psychological stress that the important change of the body would determine. In this context, the therapy has been identified as improving some psychological functioning such as decreased depression and improved global functioning [42]. In a recent study, carried out on 89 subjects undergoing the block therapy between 9 and 16 years, a reduction in suicidal ideations, psychological distress, binge drinking and drug abuse was reported [43]. Another paper highlighted the importance of a multi-disciplinary team in managing these complex patients. In particular, thanks to a correct multi-disciplinary management, the disappearance of the GD/GI in these subjects is achieved. However, an adequate diagnostic phase is needed, proper management of the pubertal block, in addition to the subsequent sex hormone treatment and gender assignment surgery [44].

If the block therapy had started in the later stages of puberty, some signs would become irreversible, such as the breast enlargement, or the voice masculinization. For all these reasons, it is essential to start during the first pubertal stages.

It should also be taken into account the aspect related to the health economy, since the early intervention during the first pubertal stages, allows to avoid several multiple (expensive) surgeries [45]. In this respect, in those subjects for which GnRHa could be too expensive, or not covered by health insurance plans, it is also possible to exploit the suppressive effect of progestins (oral or injectable progestin formulations) [19].

#### Short and long-term consequences

Therapy with GnRHa is almost always tolerated without any special problems. Some subjects may experience skin reactions at the site of the injection, such as redness and pain [46]. Sex steroid reduction could lead to the appearance of emotional lability and mood changes [39]. A single study reported arterial hypertension as an adverse effect in three subjects belonging to a cohort of 138, that however disappeared once the end of GnRHa therapy [47].

To date, long-term longitudinal data are not yet available, therefore it is not possible to clearly document both biological and psychosocial changes over time.

Considering the bone mineralization, few data are available on the effect of GnRHa. In a recent study, a retrospective review of 70 subjects aged 12–14 years was done, evaluating the bone mineral density (BMD) and the bone mineral apparent density (BMAD) during the GnRHa therapy, through annual DXA scans. The Authors documented, after the start of the therapy, a sudden drop in BMD and BMAD z-scores, a slight reduction in the BMD/BMAD z-scores ratio during the second year, but an overall absence of significant changes in both parameters after three years [48].

Another recent paper reported the short and medium-term outcomes of a prospective cohort of 44 adolescents under GnRH analogues, for a time span of 1–4 years. They analysed both spine and hip BMC and BMD through DXA scans, at 12, 24 and 36 months after the start of the therapy, demonstrating even an improvement of lumbar BMC and BMD after 24 months, indicating greater bone strength. However, BMD z-score fell consistent with delay of puberty [49].

In an observational and prospective study published in 2020, which took into account as main outcome measures the BMAD and the BMAD z-scores, 51 transgirls and 70 transboys were treated with GnRHa; furthermore, of them, 36 transgirls and 42 transboys received also the gender-affirming hormones. At the start of GnRHa treatment, aBMD and BMAD values were within the normal range, but in transgirls, the mean BMAD z-scores were below the population mean. During two years of GnRHa, the study reported a stabilization or a small decrease of BMAD, while BMAD z-scores decreased in all groups. However, the Authors found an important impact of the gender-affirming hormones, since 3 years of combined administration with GnRHa managed to determine a significant increase of BMAD in both groups and of BMAD z-scores only in transboys, whereas it stood below zero in transgirls, despite 3 years of estrogen treatment. The authors concluded the work by highlighting the not certain correlation between the data they found, and the possibility of adverse bone outcomes in the future [50].

The decline of aBMD and BMAD z-scores during GnRHa were substantially in line with other studies [36, 51, 52].

Some studies investigated changes in body composition. Klaver and colleagues examined, through a retrospettive designe, the change in body shape and body composition during GnRHa in 71 AMAB and 121 AFAB, demonstrating, in AMAB subjects, an increase fat ratio, whereas AFAB persons had decreased fat compared to cis-gender peers [53]. Schagen et al. reported the GnRHa effect of increasing in fat mass and decreasing in lean body mass percentage [37], while no change was observed in the above-cited Klink’s study [51].

Another important feature related to the use of GnRHa is the fertility issue. Adolescents that undergo puberty blockade, invariably display a scarce maturation of the gametes, as happens in hypogonadism. In addition to this aspect, there is the scant attention that the subjects with GD/GI shows towards this topic, given the psychological distress related to the condition, associated with the anxiety of wanting to transit to a more congenial body, as fast as possible. In male to female subjects, the only possibility is the cryopreservation of testicular tissue, given that, at Tanner stage 2, only 20% of transgender girls will have begun spermatogenesis. In the case of a blockage in later stages, it would be possible the collection of mature sperm via ejaculation, but the problem of the appearance of secondary sexual characters would occur [54]. In female-to-male subjects, the situation is quite similar: ovarian tissue cryopreservation is the only option available if the follicular stimulation is ineffective, as happens in the first Tanner stages (prepubertal ovaries). On the contrary, during later stages, it would be possible the oocyte cryopreservation, as done in oncological patients [55, 56].

### Fertility preservation

The current management of transgender involves therapies that could induce infertility [57]. Professional organisations, such as the Società Italiana, Genere, Identità e Salute (SIGIS), Società Italiana di Andrologia e Medicina della Sessualità (SIAMS), and Società Italiana di Endocrinologia (SIE), recommend counselling regarding the potential impact of medical gender-affirming treatments on fertility before starting transition and the available methods for oocyte, sperm, or embryo cryopreservation [58]. Gamete cryopreservation is the most established method of fertility preservation in adult transgender individuals. Oocyte [59] and sperm [60] cryopreservation are feasible and effective methods to preserve fertility for future biological parenting and should be counselled especially for adolescence transgender.

#### Oocyte cryopreservation

Oocyte cryopreservation, it is a clinically-established method that requires a 2 weeks daily hormonal stimulation for follicular development, with invasive transvaginal ultrasound examinations and egg pick-up at the end of hormonal stimulation. This technique is a viable option for transgender youth assigned females at birth who undergo GAHT and/or GAS. Regarding therapy, to date we know that GnRHas have an impact on gamete maturation but do not cause permanent damage to gonadal function: if GnRHas is suspended, oocyte maturation should resume [19]. Similarly, it has been shown that young transgender men who discontinue testosterone are able to recover restoration of normal ovarian function with oocyte maturation and achieve a natural conception [61]. But to date, no prospective studies have evaluated the effect of long-term hormone therapy on fertility, so it is important to consider options for preserving fertility. On the other side, if transgender men undergo oophorectomy, cryopreservation of ovarian tissue may also be an option. However, this method will require auto transplantation of the tissue in the future, or a further establishment of methods such as in vitro follicle growth of oocytes obtained from the tissue, which are still under development [62]. In a cross-sectional study on transgender men seeking pregnancy, 88% used their own cryopreserved oocytes before hormonal transition after the age of 17 [63]. A very recent case series from a single fertility centre of 44 adolescent transgender men reported that the majority were testosterone-naive (71%, 25/35), and had not pursued gender-affirming surgery (86%, 30/35), 57% underwent oocyte cryopreservation with a median of 22 oocytes retrieved and 15 mature oocytes cryopreserved [64]. In a case-report of three transgender men undergoing oocyte cryopreservation before transition, one of them cryopreserved during adolescence: a total of 21 oocytes were retrieved and 17 mature oocytes were successfully cryopreserved with vitrification [65]. In another case-report of a 16 years old transgender male, five oocytes were retrieved and four were cryopreserved after induction of oocyte maturation with recombinant hCG [66]. Gamete quality should also be considered given the young age of adolescent transgender men: fertility preservation of oocytes during puberty or adolescence has indeed a lower success compared with early adulthood cryopreservation [67]. Nonetheless, compared with age-matched cis-gender females, adolescent transgender men have an excellent response to ovarian stimulation before initiating hormonal stimulation [68] and the majority of cryopreserved oocytes cells after long-term androgen treatment are vital [69]. In transgender patients, oocyte cryopreservation is preferably performed before the gender-affirming hormone treatment, but even after long-term exposure to testosterone, adult transgender men undergoing cryopreservation have a comparable outcome of embryo success compared with age-matched fertile cis-gender oocytes donors [70].

#### Semen cryopreservation

On the other hand, the main method of fertility preservation for transgender women is cryopreservation of semen. Significant alteration of sperm parameters, as well as a high risk of azoospermia, has been previously observed in transgender women who initiated gender affirmation treatment [71]. Barnard et al. described an impairment of sperm production up to 4 months after treatment discontinuation. Therefore, young transgender females who have started treatment may require discontinuation for several months to allow resumption of spermatogenesis [72]. The required duration of a therapeutic window for recovery of normal spermatogenesis is not known. At least one spermatogenetic cycle is probably required [72], but it also depends on the duration, dose, and nature of the hormones, as well as individual factors [60]. However, even if sperm production is maintained, there are concerns about the potential impact of hormone treatments on sperm quality, such as epigenetic marks, that may compromise embryonic development and offspring health [60]. Therefore, it is particularly important to offer fertility preservation prior to the initiation of treatment.

Some recent studies have reported that fertility is not a priority for transgender youth [73, 74] and it is observed low adherence to fertility preservation [75–77] with less than 5% of transgender youth pursue cryopreservation [73, 74]. Other studies reported fertility preservation rates widely variable in youth from < 5% to 40% [19]. However, there is an inconsistency between the number of transgender adolescents who wish to have children and those who pursue fertility preservation [73, 77, 78]. The reasons for this difference are related to social, financial and biological implications that complicate the decision-making process toward future biological parenthood for transgender youth.

The most cited barriers are cost, which can be particularly prohibitive in countries where financial assistance is limited or absent [76, 79] and inadequate access to medical care [78]. For transgender patients we also find unique barriers that may affect fertility preservation utilization rates, such as gender dysphoria, discomfort with gamete retrieval procedures, and concerns about delayed medical transition [73, 76]. Regarding dysphoria and discomfort in gamete retrieval, as transgender males consider transvaginal oocyte retrieval invasive [75] and pregnancy as dysphoric idea [78], similarly, transgender females feel that masturbation for sperm retrieval can cause discomfort [76]. Some studies reported that more transgender women than transgender men have completed fertility preservation [75, 76]. This finding is not surprising given that the cryopreservation of semen is cheaper than cryopreservation of oocytes and female-to-male transition is more invasive due to the methods it requires [79]. The desire for parenthood is also influenced by the sex of their future partners [73, 80]. In addition, for transgender minors, parents may have an impact in the decision on fertility preservation of trans youth. Although many transgender adolescents feel uncomfortable collecting gametes, they cryopreserve only at the behest of their parents [57]. However, Persky et al. showed that parents tend to be understanding about their children's decisions [81] and it is not important for them to have biological grandchildren [73, 81]. All these barriers hinder the fertility preservation and justify the data showing that most young transgender say they want to become parents in the future, but most do not plan to have a biological child. Most of them prefer adoption [57, 72, 81] and a smaller number were open to surrogacy or gamete donation [73, 75].

The absence of such guidelines is potentially another obstacle for providers. Improving and standardizing clinical practice and counselling could help transgender youth in decision making and avoid regret in the future. Clinicians suggested that having a multi-disciplinary team within the same clinic can reduce barriers to access and improve the quality of care through collaboration of different specialists [83]. In addition, the timing of discussion of fertility preservation should be adjusted for each patient [82, 84]. Furthermore, clinicians recognized that it might be necessary to provide written information to allow patients and their families time to think. This could have advantages such as standardizing the information to be given and preventing them from inquiring elsewhere with the risk of being inaccurately informed [82]. Finally, it might be useful to personalize the approach and discuss these complex issues with younger children in an appropriate way [82, 83, 85]. Johnson et al. reported that there is a need to develop a decision aid programme that can be modulated according to each patient's clinical situation [86]. Developing a decision aid programme improve patient-clinician communication, facilitate adolescent-parent conversation, and minimize regret.

## Conclusion

**Fig. 1:**
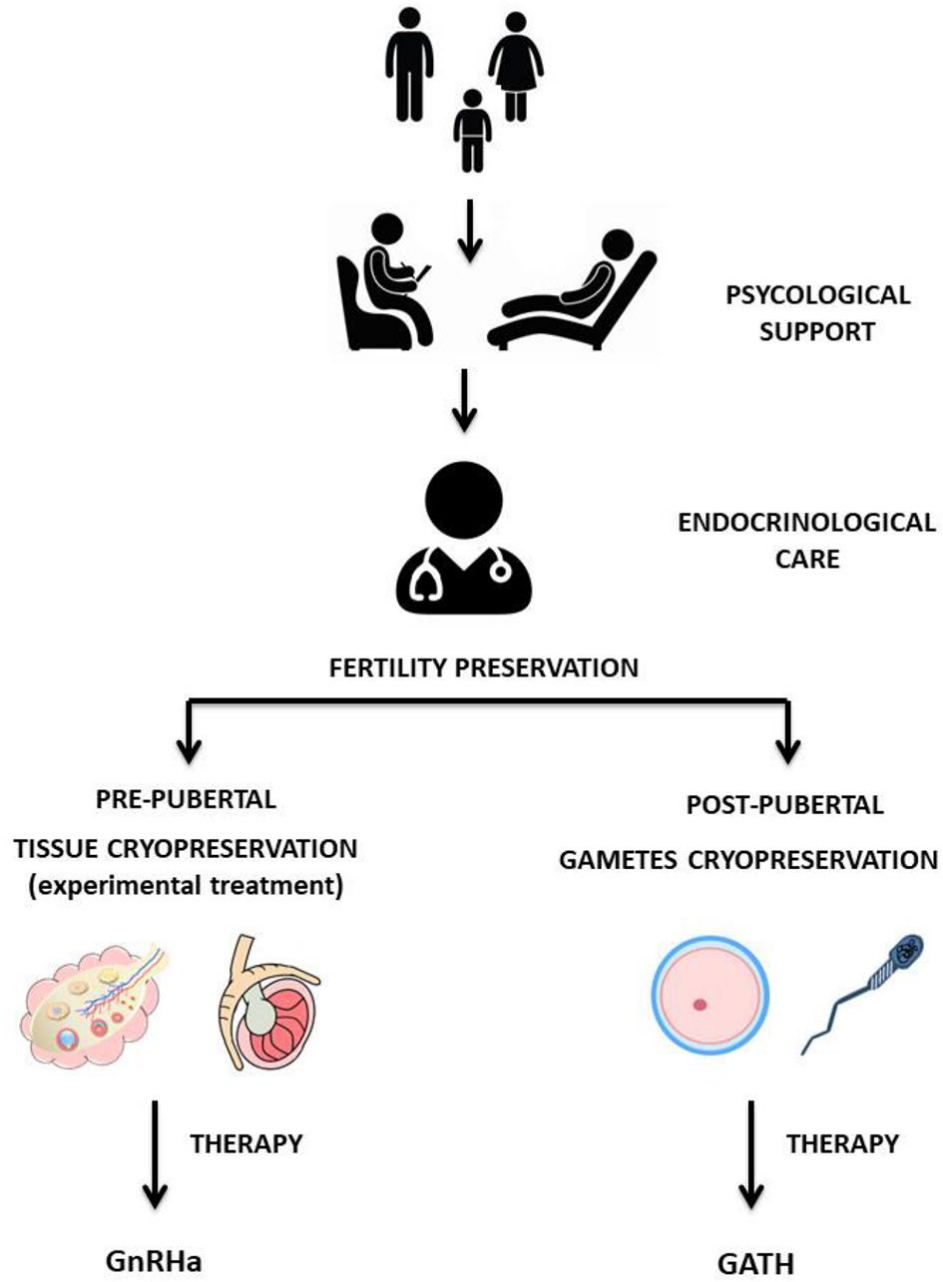
Diagnostic and therapeutic management of gender incongruence

Based on this scientific evidence and given the sensitivity of the topic, the recent version of the “Standard of Care for the Health of Transgender and Gender Diverse People” recommends health care providers to inform transgender youth about the known effects of hormone therapies/surgery on future fertility. The potential effects of therapies on gametes are not well studied; in particular, we do not know yet the timing of the spermatogenesis and ovogenesis recovery. Moreover, more studies are necessary to indicate the impact of therapy on gametes quality after the block of the therapy. For these reasons it is recommended to inform all youth about possible fertility preservation options (both established and experimental). In addition, it emphasizes the importance of the multi-disciplinary team that can provide adequate information to patients and refer them to specific clinics to initiate cryopreservation services before offering medical and/or surgical interventions that could compromise fertility (Fig. 1) [19].

## Data Availability

No data or material to share.

## Code availability

No code to share.
